# Efficacy of ibuprofen in musculoskeletal post-traumatic pain in children: A systematic review

**DOI:** 10.1371/journal.pone.0243314

**Published:** 2020-12-03

**Authors:** Niccolò Parri, Simone Lazzeri

**Affiliations:** 1 Department of Emergency Medicine and Trauma Center, Meyer University Children’s Hospital, Florence, Italy; 2 Department of Orthopaedics and Traumatology, Meyer University Children’s Hospital, Florence, Italy; University of Arizona College of Medicine, UNITED STATES

## Abstract

Musculoskeletal (MSK) injuries are one of the most frequent reason for pain-related evaluation in the emergency department (ED) in children. There is still no consensus as to what constitutes the best analgesic for MSK pain in children. However, ibuprofen is reported to be the most commonly prescribed analgesic and is considered the standard first-line treatment for MSK injury pain in children, even if it is argued that it provides inadequate relief for many patients. The purpose of this study was to review the most recent literature to assess the efficacy of ibuprofen for pain relief in MSK injuries in children evaluated in the ED. We performed a systematic review of randomized controlled trials on pharmacological interventions in children and adolescents under 19 years of age with MSK injuries according to the Preferred Reporting Items for Systematic Reviews and Meta-Analyses (PRISMA) statement. The primary outcome was the risk ratio for successful reduction in pain scores. Six studies met the inclusion criteria and provided data on 1028 children. A meta-analysis was not performed since studies were not comparable due to the different analgesic treatment used. No significant difference in term of main pain score reduction between all the analgesics used in the included studies was noted. Patients who received oral opioids had side effects more frequently when compared to children who received ibuprofen. The combination of effect on pain relief and tolerability would suggest ibuprofen as the initial drug of choice in providing relief from mild-to-moderate MSK pain in children in the ED. The results obtained in this review and current research suggest that there’s no straightforward statistically significant evidence of the optimal analgesic agent to be used. However, ibuprofen may be preferable as the initial drug of choice in providing relief from MSK pain due to the favorable combination of effectiveness and safety profile. In fact, despite the non-significant pain reduction as compared to children who received opioids, there are less side effect associated to ibuprofen within studies. The wide range of primary outcomes measured in respect of pain scores and timing of recorded measures warrants a future standardization of study designs.

## Introduction

Musculoskeletal (MSK) injuries, including sprains, strains and fractures, are one of the most frequent reasons of pain-related evaluation in the emergency department (ED) in children [1]. Indeed, fractures alone constitute between 10% and 25% of all injuries [2] and it is estimated that between one fourth and one half of children will sustain a fracture before 16 years of age [3, 4]. Studies have repeatedly demonstrated that analgesia is suboptimal in the pediatric population especially in MSK trauma [5–7] and it has been recognized that, besides the fact that today the concept of access to pain management has been accepted and incorporated it into key human rights reports by the United Nations and regional human rights bodies [8], inadequate pain treatment determines significant short- and long-term consequences in children [9] such as slower healing [10] anxiety and hyperesthesia [11], and fear of medical care [12].

Reasons for this surprisingly poor management of pain include a lack of evidence-based guidelines and concerns about risks associated with analgesics prescription [9] although pediatric and emergency societies endorse appropriate treatment of pain as a key part of clinical care [13–16].

Nonsteroidal anti-inflammatory drugs (NSAIDs) alone or in combination with other drugs, are the most commonly used analgesics in the ED management of children with MSK pain [10, 17–19].

A Cochrane review of postoperative pain in adults concluded that NSAIDs are effective, and they are normally prescribed to adult patients for various types of pain [20]. The same evidence is not available in children and there is still no consensus as to what constitutes the best analgesic for MSK pain in this population.

A recent systematic reviewed assessed pharmacological and non-pharmacological interventions for children aged 0–18 years with musculoskeletal injury and concluded that no specific analgesic agent or intervention could be identified as the optimal choice in the ED [18].

However, ibuprofen is reported to be the most commonly prescribed analgesic and is considered the standard first-line treatment for MSK injury pain in children [10, 21–24], even if it is argued that it provides inadequate relief for many patients [21].

With this in mind, we aimed to review the current literature focusing on ibuprofen in order to assess its efficacy in pain relief in MSK injuries in children evaluated in the ED and try to clarify if its preferential use is justifiable.

## Materials and methods

A systematic review of randomized controlled trial on pharmacological interventions in children and adolescents under 19 years of age presenting at the ED after having sustained a MSK injury.

A systematic search of PubMed, EMBASE and the Cochrane Central register of Controlled Trials (CENTRAL) was conducted on July, 2019 according to the Preferred Reporting items for Systematic Reviews and Meta-Analyses (PRISMA) statement [25]. The search was limited to published articles written in English and to the last 25 years (1995/01 to 2020/10).

Although a protocol was not registered, all stages of the PRISMA search (including eligibility criteria) and all outcome and analysis were planned before their execution. This search was independently conducted by 2 authors for internal validity.

Studies were identified using search combinations or a combination of words. The search strategy as well as the keywords and the search details are reported in the S1 Table.

On a first screening stage, duplicates were removed, and all identified studies were evaluated by title or abstract. The authors independently screened titles and abstracts using a standardized form with predefined eligibility criteria. Studies that were considered eligible were assessed in full-text independently to determine whether or not they met inclusion criteria. Discrepancies were resolved by consensus. Studies with adult and pediatric participants were included if pediatric specific data could be deduced. Studies related to fracture reduction or to pharmacological pain treatment related to other intervention were excluded. Studies related to MSK injuries associated with other primary skeletal conditions such as inflammatory disease or other systemic conditions were also excluded. Studies selected after this procedure were evaluated for methodological quality and appropriateness. Assessment of methodological quality in studies that met the inclusion criteria was graded using the Cochrane Collaboration’s Risk of Bias assessment tool [26]. Studies were scored as high, low or unclear risk of bias based on random sequence generation, allocation concealment, blinding of participants and personnel, blinding of outcome assessment, incomplete outcome data, selective reporting, and other issues of possible bias. The primary outcome was the risk ratio (RR) for successful reduction in pain scores. There was no need for ethical approval due to the nature of the study. The need for approval was waived by the local Clinical Trial Center.

## Results

**Fig 1:**
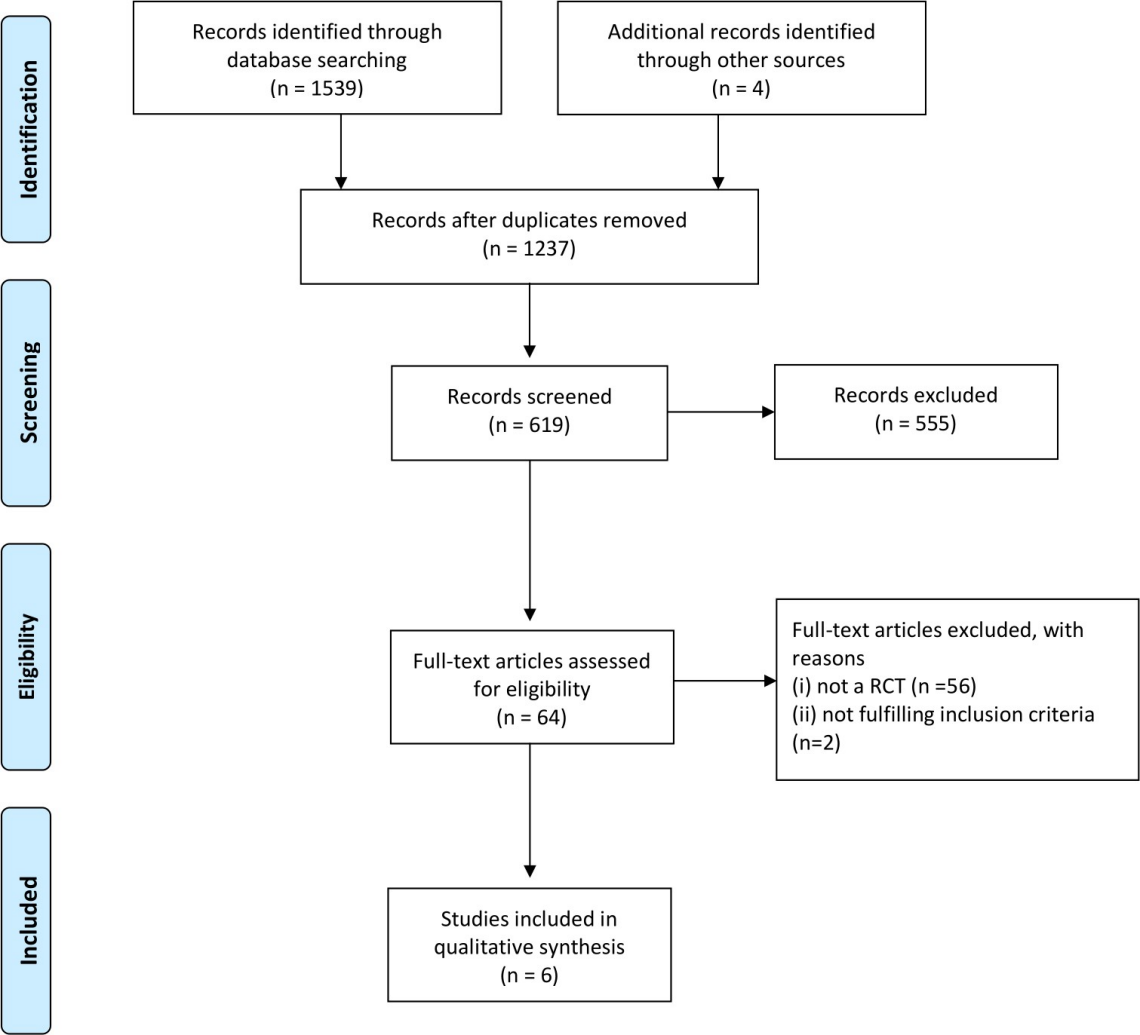
PRISMA flow diagram. RCT randomized controlled trial.

This study’s PRISMA search is summarized in Fig 1.

Of the 1539 identified articles, 64 were assessed for eligibility. Eight randomized trials had ibuprofen as a single analgesic or combined with another drug or a placebo [19, 21, 23, 27–31]. Two of these studies reported on pain management of children following acute fractures in an outpatient setting and were therefore excluded from the analysis [19, 27]. In the 6 remaining studies, which considered ibuprofen as an analgesic in the ED, the pooled number of children included was 1028 [21, 23, 28–31]. Four studies [23, 28–30] reported results as the mean pain score (MPS) difference and two [21, 31] as a change in the faces pain scale (FPS) or visual analog scale (VAS) score from baseline to pre-defined timing. Study characteristics are summarized in Table 1.

**Table 1:** Study characteristics.

Study	Year	Design	Intervention*	Outcome	Participants	Mean age (years)	Results
Koller et al. [31]	2007	Randomized, double-blind clinical trial	Group 1: 22 oxycodone 0.1mg/Kg (max 10mg) + placebo	Change in the FPS score at 120 minutes after analgesic administration	66	11.3	No difference in analgesic effectiveness
			Group 2: 22 ibuprofen 10 mg/Kg (max 800mg) + placebo Group 3: 22 oxycodone 0.1mg/Kg (max 10mg) + ibuprofen 10 mg/Kg (max 800mg) +				
Clark et al. [21]	2007	Randomized, double-blind controlled clinical trial	Group 1: 109 acetaminophen 15mg/Kg (max 650mg)	Change in patient’s self-reported pain on VAS at 60 minutes after analgesic administration	336	12	ibuprofen group had a greater improvement in pain score than those in the codeine and acetaminophen groups
			Group 2: 107 oral ibuprofen 10 mg/Kg (max 600mg) Group 3: 109 codeine 1mg/Kg (max 60mg)				
Friday et al. [28]	2009	Randomized, double-blind equivalence trial	Group 1: 34 ibuprofen 10mg/Kg	Difference in MPS>2cm on CAS at 40 minutes after analgesic administration	66	10.6	No difference in analgesic effectiveness
			Group 2: 32 acetaminophen + codeine 15mg/Kg + 1mg/Kg (max 60 mg codeine)				
Le May et al. [29]	2013	Randomized, double-blind, placebo-controlled, clinical trial	Group 1: 41 ibuprofen 10mg/Kg + codeine 1mg/Kg (max 60mg codeine)	Difference in MPS of 20mm on VAS at 90 minutes after analgesic administration	81	11.2	No significant difference
			Group 2: 40 ibuprofen 10mg/Kg (max 600mg) + placebo				
Poonai et al. [30]	2014	Randomized, parallel group, blinded superiority	Group 1: 66 morphine 0.5mg/Kg (max 10mg)	Change in pain score FPS-R at 30 minutes after analgesic administration	134	10.7	No significant difference
			Group 2: 68 ibuprofen 10mg/Kg (max 600mg) + placebo				
Le May et al. [23]	2017	Randomized, double-blind, placebo-controlled trial	Group 1: 91 morphine 0.2mg/Kg (max 15mg) + ibuprofen 10mg/Kg (max 600mg)	Difference in MPS<30mm on VAS at 60 minutes after analgesic administration	456	11.9	No significant difference
			Group 2: 188 morphine 0.2mg/Kg (max 15mg) + placebo				
			Group 3: 177 ibuprofen 10mg/Kg (max 600mg) + placebo				

*all drugs were administered orally

MPS mean pain score, CAS Color Analog Scale, FPS-R Faces Pain Scale-Revised, VAS Visual Analog Scale

**Fig 2:**
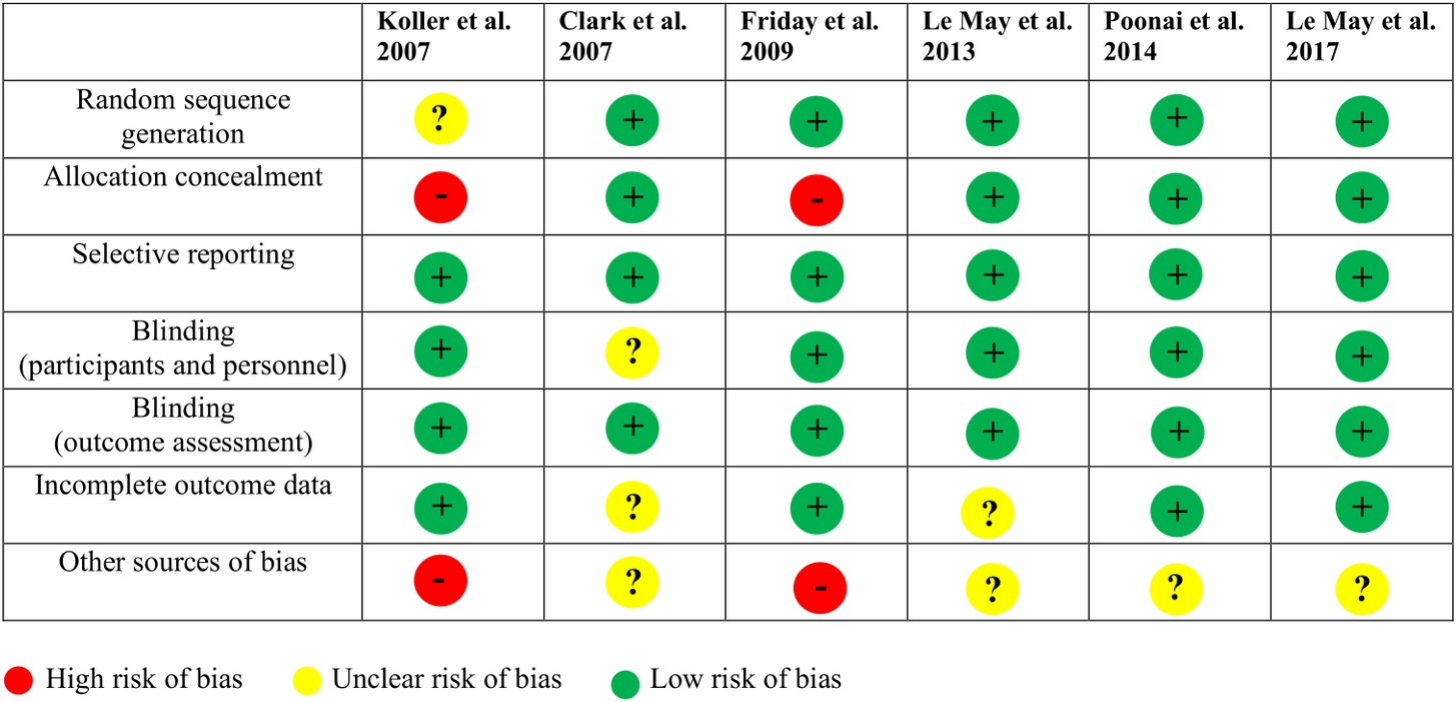
Risk of bias assessment.

The compared analgesics were: acetaminophen to ibuprofen to codeine [21], acetaminophen/codeine versus ibuprofen [28], ibuprofen/codeine to ibuprofen/placebo [29], oral morphine to ibuprofen [30], oral morphine/ibuprofen to oral morphine/placebo to ibuprofen/placebo [23] and oxycodone/placebo to ibuprofen/placebo to oxycodone/ibuprofen [31]. Pain was assessed using different pain scales: Visual Analog Scale (VAS) [23, 29, 31], Color Analog Scale (CAS) [28], Faces Pain Scale-Revised (FPS-R) [30] and Faces Pain Scale (FPS) [21]. The primary outcome in 4 studies [23, 28–30] was the MPS difference and in 2 [21, 31] the change in patient’s pain after analgesic administration. The MPS difference indicative of analgesic efficacy varied across studies depending on the pain scale used. Friday et al. [28] used a MPS difference > 20 mm on CAS as an adequate pain relief, Le May et al. [29] used 20 mm on VAS while Poonai et al. [30] considered a change in pain score on FPS-R <30 mm. Le May et al. [23] identified pain intensity < 30 mm on the VAS scale as target of adequate pain relief. Koller et al. [31] considered a change of the FPS score while Clark et al. [21] used a difference on VAS score from baseline, both after analgesic administration, as a measure of adequate pain relief. These differences in pain scores were registered at different time after receiving the analgesic across the studies: 30 minutes [30], 40 minutes [28], 60 minutes [21, 23], 90 minutes [29] and 120 minutes [31]. In all of the included studies, there were no control groups receiving no analgesia, as it would have been considered ethically unacceptable. Fig 2 presents the risk of bias in the included studies according to the Cochrane Collaboration’s Risk of Bias tool [26].

The risk of bias was assessed by two independent raters (SL and MG) and disagreement was resolved through consultation with the first author (NP).

Overall, all the included studies, reported that a randomization sequence was adequately generated [21, 23, 28–31]. In 4 studies, allocation was adequately concealed [21, 23, 29, 30]. Two studies presented a high risk of bias due to inadequate concealment of allocations prior to assignment [28, 31]. Selective reporting bias was found low in all included studies [21, 23, 28–31].

Blinding of participants and personnel was adequate in 5 out of 6 studies [23, 28–31]. Blinding of outcome was adequately performed in all studies [21, 23, 28–30].

Incomplete outcome data were adequately addressed in 4 out of 6 studies [23, 28–30].

Two studies [28, 31] displayed a high risk of bias (other biases) due to enrollment criteria (triage pain score of 5) [28] and the non-consecutive enrollment that may have selected a population where pain was inadequately treated by the oral route [28, 31]. Second, one study [31] excluded a sample of patience from the convenience sample enrolled which may have differed from the sample considered in the final analysis despite the use of randomization. In addition, patients included in these study, received splints [28] and ice packs [28, 31] when appropriate irrespective of randomization and unrelated to study drugs. Of note, in the systematic review of Le May et al. [18] this information was not perceived as a high risk of bias. Nonetheless ice may have reduced pain or either may have also been a confounder by erroneously raising the pain is some subjects as it creates a noxious stimulus resulting in the perception of greater pain.

Structured data of 5 studies were available to calculate risk ratios (RR). Researchers of one study [31] were contacted to obtain the necessary details to calculate the RR without response. The RR of all the comparisons between different therapy was not significant for all analgesics or combination of analgesics (Table 2).

**Table 2:** Risk ratios of the comparison between different analgesic treatment.

Study	Year	Comparison	RR (95%CI)	*P*
Clark et al. [21]	2007	codein to acetaminophen	0.98 (0.91–1.05)	0.63
		acetaminophen to ibuprofen	1.01 (0.94–1.09)	0.63
		codein to ibuprofen	1.00 (0.92–1.08)	1.00
Friday et al. [28]	2009	acetaminophen + codeine to ibuprofen	1.00 (0.60–1.66)	0.99
Le May at al. [29]	2013	ibuprofen + codeine to ibuprofen + placebo	0.76 (0.45–1.29)	0.31
Poonai et al. [30]	2014	oral morphine to ibuprofen	0.80 (0.39–1.65)	0.65
Le May et al. [23]	2017	ibuprofen + placebo to oral morphine + placebo	1.12 (0.78–1.62)	0.57
		ibuprofen + placebo to oral morphine + ibuprofen	1.10 (0.76–1.59)	0.67
		oral morphine + placebo to oral morphine + ibuprofen	0.97 (0.71–1.34)	0.90

RR Risk ratio, CI confidence interval.

Clark et al. [21] compared acetaminophen, ibuprofen, and codeine in 3 different cohorts. Differences between groups were assessed by measuring the change in patient’s pain through the VAS score at 60 minutes after receipt of the study medication. The RR was not significant for either comparison (RR: 1.01; 95%CI: 0.94–1.09; *P* = 0.63 for the comparison of acetaminophen vs. ibuprofen; RR: 0.98; 95%CI: 0.91–1.05; *P* = 0.63 for the comparison of codeine vs acetaminophen; RR: 1.00; 95%CI: 0.92–1.08; *P* = 1.00 for the comparison of codeine vs ibuprofen.

Friday et al. [28] compared the analgesic effectiveness of acetaminophen-codeine with that of ibuprofen. The primary outcome was measured was the difference in CAS scores changes measured at 40 minutes in patients with acute traumatic extremity pain. The RR was not significant towards either group (RR: 1.00; 95% CI: 0.60–1.66; *P* = 0.99).

Poonai et al. [30] compared analgesic efficacy between orally administered morphine and ibuprofen. In this study, pain intensity evaluation was performed 30 minutes after drug administration. The primary outcome was change in pain scores using FPS-R. The RR was not significant to either group (RR: 0.80; 95%CI: 0.39–1.65; *P* = 0.65).

Le May at al. [29] compared the efficacy of a combination of codeine and ibuprofen versus ibuprofen and placebo. The primary outcome was difference in pain over time between groups assessed with the VAS. The RR was not in favor to either group (RR: 0.76; 95%CI: 0.45–1.29; *P* = 0.31). Le May et al. [23] compared in a more recent study morphine and ibuprofen to either morphine alone or ibuprofen alone. Differences between groups were assessed by measuring the proportion of participants achieving pain intensity scores >30mm on the VAS scale 60 minutes after medication administration. Again, RR was not significant for either comparison (RR: 1.12; 95%CI: 0.78–1.62; *P* = 0.57 for the comparison of ibuprofen and placebo vs. oral morphine and placebo; RR: 1.10; 95%CI: 0.76–1.59; *P* = 0.67 for the comparison of ibuprofen and placebo vs. oral morphine and ibuprofen; RR: 0.97; 95%CI: 0.71–1.34; *P* = 0.90 for the comparison of oral morphine and placebo vs. oral morphine and ibuprofen).

Finally, in one study with missing data to calculate the RR, ibuprofen was found to be equivalent to oxycodone [31].

We were not able to combine between studies and this also precluded a meta-analysis as each study compared different drugs.

## Discussion

Children are very frequent victims of trauma [2–5] making their presentation to the ED following a MSK injury very common [1]. Unfortunately, current pain management is widely recognized to be frequently insufficient, possibly because of uncertainty on which medications to prescribe, to the lack of clear and well established guidelines, to fear of adverse effects [8]. Determining the adequate analgesic agent for children with acute MSK pain can be a complex decision, especially in emergency settings and can be influenced by multiple factors including patient age, ability to swallow pills, pre-existing side effect with other drugs.

In this study, we reported on the efficacy of ibuprofen as sole therapy or associated with other drug, as emergency medication in children evaluated in the ED for MSK trauma. We focused on this specific drug since it appears to be the most frequently prescribed medication and is often suggested as first-line treatment [10, 21–24].

A previous review of this subject included trials of nonpharmacological, physical, and pharmacological (e.g., opioids, non-opioids, and non-steroidal anti-inflammatory medications) interventions in under 19 years of age presenting to the ED with MSK injury [18].

This review included eight randomized controlled trials [21, 28, 29, 32–36] assessing analgesics via different routes with different drugs. Four of these studies considered ibuprofen as an analgesic medication [21, 28, 29, 32]. One of these [32], included 73 children with limb injuries who were randomized to intranasal (IN) ketamine + ibuprofen or IN fentanyl + ibuprofen. This article was not considered for our systematic review because the use of IN drugs associated to ibuprofen which results in the impossibility of evaluation of the efficacy of ibuprofen as single analgesic. The conclusion of this systematic review of Le May [18], is that no one specific analgesic agent has been clearly identified as the optimal choice in the ED for all scenarios of pain related to pediatric MSK injuries. This result underscore the need for further evidence for pain treatment of children presenting to the ED with a MSK injury.

The main goal of our systematic review was to address specifically on of ibuprofen as analgesic for acute MSK injury in children presenting to the ED by adding evidence from more recent trials [23]. We excluded studies that compared pain management for post-discharge pain and studies which did not considered ibuprofen as a single analgesic.

This review included 6 randomized controlled trials [21, 23, 28–31] published in the past 25 years, assessing analgesics exclusively via oral route. Three of the included studies had been already object of a previous systematic review [18].

Based on our review of the current evidence, in the pediatric population assessed in the ED for MSK trauma, there’s no straightforward statistically significant evidence of the optimal analgesic agent to be used. Nevertheless, some observation pertinent to the examined trials have to be considered.

In the study by Clark et al. [21] ibuprofen provided greater pain relief from acute MSK injuries than codeine or acetaminophen. Patients receiving ibuprofen were also more likely to obtain adequate analgesia. Although ibuprofen was more efficacious in providing adequate analgesia, only 52% of children receiving ibuprofen could be defined as having received an “adequate analgesia” (defined as <30mm on a 100mm Visual Analog Scale) after 60 minutes from the analgesic administration.

Koller et al. [31] reported that oxycodone was no more effective than ibuprofen or the combination of the two drugs for the management of orthopedic injury-related pain. The individual groups of treatment of this study were small, and it is possible that a larger study would find a significant difference between treatments.

In the study by Friday et al. [28] equivalent analgesic effectiveness of ibuprofen and acetaminophen-codeine combination as was demonstrated by a similar decrease in CAS score at 40 minutes in patients with acute traumatic extremity pain. This study challenged the reputed superiority of mild narcotic over ibuprofen for acute MSK pain in children. However, in this study the maximum prescribed dose for ibuprofen was 400 mg even for patients heavier than 40 kg. Also, since one of the entry criteria was a high score on the triage pain evaluation, a population with conditions non-treatable with oral analgesia might have been selected. Adverse effects were reported to be minimal.

In the study by Poonai et al. [30] again, no significant difference in analgesic efficacy between orally administered morphine and ibuprofen was documented. In this study, however, pain intensity evaluation was performed 30 minutes after drug administration, which is 30 minutes early of peak effect of both drugs. Nevertheless, both morphine and ibuprofen determined a decrease in pain scores at each dose administration but significantly more participants in the morphine group had adverse effects (56% versus 31%), the most common of which was drowsiness.

Le May et al. [29] compared the efficacy of a combination of codeine and ibuprofen versus ibuprofen and placebo. Although statistical significance was observed on mean differences in pain scores between measurement periods from triage, the study did not reveal any difference between study groups, concluding that the addition of codeine to ibuprofen did not improve pain management in children with MSK pain. Side effects were minimal.

Finally, in the more recent trial by Le May et al. [23] again, no statistically significant difference in pain reduction could be observed when comparing ibuprofen associated to placebo to a morphine and ibuprofen association or to a morphine and placebo association, although an exception to the general finding was observed at 120 minutes after drug administration, where the MPS reduction was more evident in the group of patients who took the ibuprofen and placebo association. In this study, although no serious adverse events were recorded in either group, more children in the morphine and ibuprofen group (*P*<0.001) and in the morphine and placebo group (*P*>0.001) experienced adverse events when compared to the ibuprofen and placebo group. Nausea, abdominal pain and drowsiness were reported from 2 up to 6% in the groups who received morphine, whether associated with ibuprofen or placebo.

We feel that, if all the above factors are combined, data suggest that ibuprofen should be recommended as first choice treatment in mild-to-moderate acute MSK pain in children in the ED setting. In fact, ibuprofen never performed inferior to all tested comparisons and, matching effectiveness in pain reduction, provided a more favorable safety profile. This was documented also for gastro-intestinal side effects, were acetaminophen demonstrated a similar gastro-intestinal side effect profile [37]. For this reason, limiting short-term ibuprofen use due to concern for gastro-intestinal problems, is not supported by current evidence [6]. If one considers the potential risks associated with opioid use, the preference allowed to ibuprofen is even more obvious.

Moreover, results of previous research suggest that in the acute phase, ibuprofen might provide a faster onset of analgesia compared to other orally administered treatment [27].

It is, however, debatable if ibuprofen alone may be adequate when dealing with moderate-to-severe pain.

There are, however, some limitations inherent to this review. Statistically significant reduction of pain indicated with a lower scoring scale as target of adequate pain relief does not reflect clinical relevant pain relief. Pain scores measure something, that may not be substantial as they do not accurately identify which patients want or should receive analgesics. Their application, apart from research settings, could encourage health care providers treat numbers rather than the patient [38]. Disparity in analgesic regimen chosen and variations in reporting of efficacy prevented the pooling of the results. Also, the wide range of primary outcomes measured in respect of pain scores and timing of recorded measures warrants a future standardization of study designs. Studies did not control for a potential confounder such as limb elevation and splinting as limb elevation and splinting is likely to reduce pain and might influence pain scores. Future research should take in consideration the analysis of all the potential confounders that could limit the correct interpretation of pain reduction provided by analgesics. Finally, the review was limited to english language articles and no gray-literature was searched.

Based on our review of the current evidence, in the pediatric population assessed in the ED for MSK trauma, there is no straightforward statistically significant evidence of the optimal analgesic agent to be used. However, our review suggests that ibuprofen may be preferable as the initial drug of choice in providing relief from MSK pain due to the favorable combination of effectiveness and safety profile. In fact, despite the non-significant pain reduction as compared to children who received opioids, there are less side effect associated to ibuprofen within studies. We hope that our review will provide further evidence for rational analgesic therapy for clinician dealing with MSK trauma in the ED.

## Supporting information

S1 Table Search strategy and keywords. (PDF)
