# Supplementary material for: Efficacy of ibuprofen in musculoskeletal post-traumatic pain in children: A systematic review
Source: PLoS One. 2020 Dec 3;15(12):e0243314. doi: 10.1371/journal.pone.0243314 (PMC7714211; doi:10.1371/journal.pone.0243314)
Supplement: S1 Table — (PDF) [file pone.0243314.s001.pdf]

| |
|---|
| **Search 1** |
| **Keywords:** NSAIDs; Pediatric Trauma |
| **Search Details:** ("anti-inflammatory agents, non-steroidal"[Pharmacological Action] OR "anti-inflammatory agents, non-steroidal"[MeSH Terms] OR ("anti-inflammatory"[All Fields] AND "agents"[All Fields] AND "non-steroidal"[All Fields]) OR "non-steroidal anti-inflammatory agents"[All Fields] OR "nsaids"[All Fields]) AND (("pediatrics"[MeSH Terms] OR "pediatrics"[All Fields] OR "pediatric"[All Fields]) AND ("injuries"[Subheading] OR "injuries"[All Fields] OR "trauma"[All Fields] OR "wounds and injuries"[MeSH Terms] OR ("wounds"[All Fields] AND "injuries"[All Fields]) OR "wounds and injuries"[All Fields])) Filters: from 1995/1/1 - 2020/10/01 |
| **Results** 151 |
| **Search 2** |
| **Keywords:** NSAIDs; children; trauma |
| **Search Details:** ("anti-inflammatory agents, non-steroidal"[Pharmacological Action] OR "anti-inflammatory agents, non-steroidal"[MeSH Terms] OR ("anti-inflammatory"[All Fields] AND "agents"[All Fields] AND "non-steroidal"[All Fields]) OR "non-steroidal anti-inflammatory agents"[All Fields] OR "nsaids"[All Fields]) AND ("child"[MeSH Terms] OR "child"[All Fields] OR "children"[All Fields]) AND ("injuries"[Subheading] OR "injuries"[All Fields] OR "trauma"[All Fields] OR "wounds and injuries"[MeSH Terms] OR ("wounds"[All Fields] AND "injuries"[All Fields]) OR "wounds and injuries"[All Fields]) Filters: from 1995/1/1 - 2020/10/01 |
| **Risultati** 295 |
| **Search 3** |
| **Keywords:** Acute Pain; NSAIDs; Child |
| **Search Details:** ("Acute Pain"[Mesh] AND "Anti-Inflammatory Agents, Non-Steroidal"[Mesh]) AND "Child"[Mesh] Filters: from 1995/1/1 - 2020/10/01 |
| **Results** 10 |
| **Search 4** |
| **Keywords:** Acute Pain; analgesics; child |
| **Search Details:** ("acute pain"[MeSH Terms] OR ("acute"[All Fields] AND "pain"[All Fields]) OR "acute pain"[All Fields]) AND ("analgesics"[Pharmacological Action] OR "analgesics"[MeSH Terms] OR "analgesics"[All Fields]) AND ("child"[MeSH Terms] OR "child"[All Fields]) Filters: from 1995/1/1 - 2020/10/01 |
| **Results** 815 |
| **Search 5** |
| **Keywords:** Acute pain; NDSAIDs; Pediatric Trauma |
| **Search Details:** ("acute pain"[MeSH Terms] OR ("acute"[All Fields] AND "pain"[All Fields]) OR "acute pain"[All Fields]) AND ("anti-inflammatory agents, non-steroidal"[Pharmacological Action] OR "anti-inflammatory agents, non-steroidal"[MeSH Terms] OR ("anti-inflammatory"[All Fields] AND "agents"[All Fields] AND "non-steroidal"[All Fields]) OR "non-steroidal anti-inflammatory agents"[All Fields] OR "nsaids"[All Fields]) AND (("pediatrics"[MeSH Terms] OR "pediatrics"[All Fields] OR "pediatric"[All Fields]) AND ("injuries"[Subheading] OR "injuries"[All Fields] OR "trauma"[All Fields] OR "wounds and injuries"[MeSH Terms] OR ("wounds"[All Fields] AND "injuries"[All Fields]) OR "wounds and injuries"[All Fields])) Filters: from 1995/1/1 - 2020/10/01 |
| **Results** 13 |
| **Search 6** |
| **Keywords:** Analgesics; oral administration; fracture |
| **Search Details:** ("Analgesics/administration and dosage"[Mesh] AND "Administration, Oral"[Mesh]) AND "Fractures, Bone"[Mesh] Filters: from 1995/1/1 - 2020/10/01 |
| **Results** 20 |

| |
|---|
| **Search 7** |
| **Keywords:** Analgesics; Child; Injury |
| **Search Details:** ("Analgesics/administration and dosage"[Mesh] AND "Child"[Mesh]) AND "Wounds and Injuries"[Mesh] Filters: from 1995/1/1 - 2020/10/01 |
| **Results** 151 |
| **Search 8** |
| **Keyword:** Ibuprofen; Muscuoskeletal Injury |
| **Search Details:** ("ibuprofen"[MeSH Terms] OR "ibuprofen"[All Fields]) AND (musculoskeletal[All Fields] AND ("wounds and injuries"[MeSH Terms] OR ("wounds"[All Fields] AND "injuries"[All Fields]) OR "wounds and injuries"[All Fields] OR "injury"[All Fields])) Filters: from 1995/1/1 - 2020/10/01 |
| **Results** 30 |
| **Search 9** |
| **Keywords:** Acute Pain; Drug Therapy; Children |
| **Search Details:** "Acute Pain/drug therapy"[Mesh] AND "Child"[Mesh] Filters: from 1995/1/1 - 2020/10/01 |
| **Results** 54 |
